# From declared interest to non-participation: a feasibility study of a multi-stage testicular cancer prevention intervention among young men

**DOI:** 10.3389/fpubh.2026.1850911

**Published:** 2026-06-12

**Authors:** Małgorzata Pasek, Katarzyna Widła, Monika Łabuzek, Anna Goździalska, Małgorzata Jochymek

**Affiliations:** 1 Department of Nursing, Faculty of Medicine and Health Sciences, University of Applied Sciences in Tarnów, Tarnów, Poland; 2 Faculty of Health Sciences, Andrzej Frycz Modrzewski Krakow University, Kraków, Poland

**Keywords:** feasibility, health promotion, men's health, process evaluation, students, testicular cancer, testicular self-examination

## Abstract

**Background:**

Testicular cancer primarily affects young men, yet engagement in preventive behaviors, including testicular self-examination (TSE), remains limited. From a public health perspective, it is important not only to assess knowledge and attitudes, but also to examine whether preventive interventions can be implemented successfully under real-world conditions. This study aimed to evaluate the feasibility and implementation of a multi-stage testicular cancer prevention project conducted in an academic setting.

**Methods:**

The study was designed as a diagnostic-educational project consisting of three stages: a baseline survey, an educational component (online materials and workshops on TSE), and a follow-up survey. Approximately 600 men affiliated with the academic community were invited to participate. Process indicators included recruitment reach, participation in the educational component, and retention across study stages.

**Results:**

The baseline survey was completed by 76 participants (12.6%). No participants attended the educational workshops, and 8 individuals completed the follow-up survey (10.5% of the baseline sample). Findings from the diagnostic stage indicated low engagement in preventive behaviors and limited knowledge of correct TSE technique. Although respondents expressed interest in health-related topics, this interest did not translate into sustained participation in subsequent stages of the project.

**Conclusions:**

Limited engagement in the educational component should be interpreted not only as a study limitation, but also as an observation suggesting the presence of barriers to participation in preventive interventions delivered under real-world conditions.

The results suggest a need for interventions that are better aligned with the preferences of young men, particularly with respect to anonymity, low-threshold access, and psychologically less demanding formats.

## 1 Introduction

Testicular cancer is one of the most commonly diagnosed malignancies among young men and remains an important issue in early detection and health education. Although prognosis is generally favorable, particularly when the disease is diagnosed early and treated appropriately, delayed presentation may be associated with more advanced disease, more intensive treatment, and greater psychological burden for the patient (1, 2). Preventive and educational efforts targeted at young men therefore remain an important component of public health practice.

In this context, prevention does not refer to population-based screening in the classical sense, but rather to increasing health awareness, recognition of warning signs, and readiness to seek prompt medical advice. One of the most frequently discussed elements in this area is testicular self-examination (TSE), considered as part of body awareness and health consciousness. At the same time, acceptance of this practice, its regular performance, and practical knowledge of how to perform it correctly remain limited (3–6).

Young men represent a particularly challenging group for preventive interventions (7–9). Their contact with healthcare services tends to be less frequent than in other populations, and health behaviors are often reactive rather than proactive. In addition, issues related to intimate health may evoke embarrassment, avoidance, low perceived personal risk, and a preference for educational formats that are brief, anonymous, and do not require direct self-disclosure (3–6, 10, 11). As a result, declared openness to health education does not necessarily translate into actual participation in preventive activities.

Previous studies have shown that young men's knowledge of testicular cancer, warning signs, and TSE is variable, whereas regular engagement in preventive behaviors is generally low. The literature suggests that the effectiveness of educational interventions depends not only on the content provided, but also on the mode of delivery, perceived acceptability, psychological burden, and the extent to which the intervention is embedded in participants' everyday environment (10, 12–15). For this reason, the evaluation of prevention programs should extend beyond knowledge and attitudes to include feasibility, reach, participant retention, and implementation processes.

Feasibility assessment is particularly important in interventions delivered under real-world conditions, outside tightly controlled experimental settings. Even a well-designed intervention may have limited practical value if it fails to achieve satisfactory participation, retain participants across project stages, or ensure meaningful engagement with the educational component. From this perspective, implementation failure may also provide valuable information for the design of future health promotion strategies.

The aim of this study was to evaluate the feasibility and implementation of a multi-stage testicular cancer prevention project conducted in an academic setting.

## 2 Materials and methods

The study was designed as a multi-stage diagnostic-educational project conducted at the University of Applied Sciences in Tarnów, Poland.

The project framework was intended not only to identify the baseline level of participants' knowledge, attitudes, and preventive behaviors, but also to assess the practical feasibility of implementing educational activities related to testicular cancer under naturalistic conditions, outside a tightly controlled experimental environment.

The project consisted of three stages: a diagnostic stage, an educational component, and a follow-up stage. Stage I involved a baseline survey assessing participants' knowledge, attitudes, and behaviors related to testicular cancer prevention. Stage II comprised an educational component that included online materials and an invitation to attend practical workshops on testicular self-examination. Stage III served as a follow-up stage and was intended to assess participant retention and the feasibility of linking responses across stages. This study design made it possible to assess not only initial interest in participation, but also participants' willingness to remain involved in subsequent and potentially more demanding elements of the intervention.

Approximately 600 men affiliated with the academic community were invited to participate. This population was selected because academic settings include a substantial number of young men within the age range relevant to the epidemiology of testicular cancer. At the same time, such settings provide an opportunity to combine face-to-face recruitment with the online distribution of educational materials. Recruitment was conducted through direct in-person contact on campus, following the required organizational approvals, as well as through the university website and QR code-based materials. Participation was voluntary.

To maintain confidentiality and enable the planned linkage of responses between Stages I and III, participants were provided with a unique participation code in the form of a keychain tag. This code was entered in the survey and functioned as a pseudonymization tool without the collection of identifying data. This approach was intended to make it possible to track continuity of participation while preserving respondent anonymity.

The questionnaire included sociodemographic items and questions related to knowledge and behaviors associated with testicular cancer prevention, including TSE practice. In Stage I, standardized instruments were also used to assess selected psychological variables, including optimism (LOT-R). The Life Orientation Test-Revised (LOT-R), developed by Scheier, Carver, and Bridges and adapted for Polish populations by Juczyński, is used to assess generalized expectations of positive outcomes in adults. Optimism was included as a psychological variable potentially associated with proactive health behaviors and engagement in preventive activities. The scale contains 10 items, 6 of which are diagnostic. The instrument has acceptable internal consistency (Cronbach's alpha = 0.76) (16, 17).

The educational component included online materials, including video-based resources, as well as an invitation to attend in-person workshops using training models designed to teach TSE. The workshops were intended to serve as a more engaging element of the intervention by allowing participants not only to review educational content but also to practice the skill itself. Due to technical limitations, exposure to the online materials, such as the number of views or plays, was not recorded. This limited the ability to assess actual contact with the educational component.

Feasibility was assessed by analyzing the number of participants entering the study at baseline, participation in the educational component, and retention at follow-up. The main implementation indicators included recruitment reach, the proportion of invited individuals who completed Stage I, participation in the workshops, and the proportion of participants who completed Stage III. These indicators were intended to show the extent to which the intervention format was both feasible and acceptable under real-world conditions.

Statistical analyses were performed using IBM SPSS Statistics. Descriptive statistics were used; categorical variables are presented as frequencies and percentages, while age is reported as median and interquartile range (Q1–Q3). Project implementation indicators, including the number of participants at each stage, workshop attendance, and follow-up participation, were reported on the basis of project documentation. Survey data were analyzed using available-case analysis. For some variables, the number of observations differed due to missing data; the relevant denominator for each analysis is reported in the text and tables. Associations between selected variables were examined using appropriate nonparametric tests. Due to the feasibility-oriented and exploratory nature of the study, as well as the relatively small sample size, statistical analyses were interpreted cautiously and were used primarily to identify potential associations rather than to support definitive conclusions.

The study was conducted in accordance with the principles of the Declaration of Helsinki. Ethical approval was obtained from the Bioethics Committee of the University of Applied Sciences in Tarnów (Resolution No. 4/2023, dated June 14, 2023). Participation was voluntary; all participants provided informed consent, and data were collected in pseudonymized form using a unique participation code.

## 3 Results

A total of 76 men took part in Stage I of the study, corresponding to a participation rate of 12.6%. The mean age of participants was 25.29 years.

In the next stage, no registrations were recorded for the educational workshops on testicular self-examination, despite the scheduling of 12 workshop days and the availability of online educational materials. The follow-up survey was completed by 8 participants, representing 10.5% of the baseline sample and 1.3% of the total invited population (Table 1).

**Table 1:** Participation flow across project stages.

Project stage	Number of participants (*n*)	% of invited individuals	% of stage I participants
Invited individuals	~600	100%	–
Stage I: baseline survey	76	12.6%	100%
Stage II: workshops	0	0%	0%
Stage III: follow-up survey	8	1.3%	10.5%

Despite the use of both in-person and online recruitment strategies, participation declined markedly across subsequent stages of the project. No participation in the educational workshops was recorded, and only a small proportion of participants completed the follow-up stage (*n* = 8).

Due to technical limitations, it was not possible to monitor participants' exposure to the online educational materials, which limits interpretation of the relationship between the intervention and the observed outcomes.

A more detailed analysis was conducted for the subgroup of participants for whom Stage I data were available. The findings suggest limited engagement in preventive behaviors related to testicular cancer within the analyzed sample. Most participants did not perform regular TSE, and knowledge of the correct technique was low; only 14.47% of respondents correctly identified all elements of the procedure. At the same time, reported motivations for participation were predominantly cognitive in nature, with more than half of respondents indicating that they wanted to gain health-related knowledge (57.89%). These findings may indicate that initial participation was driven primarily by informational interest, whereas willingness to engage in activities requiring greater personal involvement appeared to be lower.

This interpretation is consistent with the decline in participation observed across later stages of the project; however, causal conclusions cannot be drawn (Table 2).

**Table 2:** Association between performing testicular self-examination and optimism level.

Variable	TSE not performed			TSE performed			U	Z	*p*	r_g_
	M	SD	M_r_	M	SD	M_r_				
LOT-R	12.77	4.23	34.20	14.75	2.94	45.88	465.50	−2.23	0.025	−0.31

M_r_, mean rank.

The analysis showed that individuals who reported performing TSE had higher optimism scores than those who did not perform TSE (M = 14.75 vs. 12.77). The difference was statistically significant (*p* = 0.025), and the effect size (rg = −0.31) may be interpreted as at least moderate. This finding indicates an association between TSE practice and higher optimism, although it does not support causal conclusions.

## 4 Discussion

The aim of this study was to evaluate the feasibility and implementation of a multi-stage testicular cancer prevention project conducted in an academic setting. The findings point to substantial challenges in maintaining participant engagement across subsequent stages of the intervention. Although some interest in participation was observed at baseline, this did not translate into involvement in the more demanding components of the project or sustained participation at follow-up. Importantly, the absence of participation in the educational intervention may reflect practical barriers to implementing preventive interventions in this population and therefore constitutes an informative implementation-related observation (2, 12–14). Low participation and retention should not be interpreted solely as methodological shortcomings, but also as indicators of limited acceptability of the intervention format in this population. In feasibility research, non-participation constitutes an important outcome, reflecting barriers to engagement that may not be observable in controlled study settings. This observation is consistent with behavioral models that emphasize the intention–behavior gap in health-related actions.

One of the most important observations is the discrepancy between declared interest in health-related issues and actual engagement in preventive action. More than half of respondents expressed a cognitive motivation for participating, namely a desire to gain health-related knowledge, yet this did not lead to participation in the more engaging elements of the project. This observation may be interpreted in the context of the intention–behavior gap described in the health behavior literature (15, 18–20). Declared interest alone does not necessarily lead to actual behavior, particularly when such behavior requires time, overcoming embarrassment, or engaging more directly with intimate health issues.

This challenge may be especially pronounced among young men. Testicular cancer prevention concerns an intimate part of the body and may therefore be associated with embarrassment, avoidance, reduced comfort with participation, and reluctance to disclose interest in the topic to others (7–9, 21). Also relevant is the low perceived personal risk of disease that is common among young adults, who often view themselves as healthy and unlikely to face serious health problems. As a result, preventive activities may be regarded as reasonable and worthwhile in principle, yet still not be undertaken in practice.

The low rate of engagement in testicular health prevention and the limited interest in educational activities observed in this study may be associated with psychosocial factors discussed in previous literature, including embarrassment, internalized masculinity norms, and reluctance to disclose vulnerability.

These findings are consistent with broader observations in the men's health literature. Previous studies have repeatedly shown that norms associated with masculinity, such as self-reliance, reluctance to show vulnerability, and avoidance of help-seeking, may reduce engagement in health-related behaviors (7–9). In the context of testicular cancer, this mechanism may be further reinforced by the intimate nature of the issue and the discomfort associated with openly discussing symptoms or body self-monitoring. Against this background, the lack of interest in practical workshops may indicate limited acceptability of this intervention format in the study population.

Another notable finding is the low level of practical knowledge related to TSE in the study group. This may suggest that general openness to health topics does not necessarily translate into the concrete skills needed to adopt preventive behaviors. From the perspective of designing educational programs, it therefore seems important not only to communicate the value of prevention, but also to identify educational formats that enhance participants' sense of safety, lower the threshold for engagement, and are not perceived as overly demanding or embarrassing (4–6, 22).

The results also suggest that the mere availability of online educational materials is not sufficient to ensure intervention effectiveness. The results suggest that the availability of online educational materials alone may be insufficient to ensure meaningful engagement with the intervention (13, 14, 23). In the present project, actual exposure to the materials could not be monitored, which limits interpretation of their potential contribution. From an implementation perspective, however, this is an important observation: in real-world interventions, it is not enough to provide educational content; it is equally important to assess whether participants actually engage with it. Without such data, it is difficult to distinguish between a situation in which the intervention was available but unattractive and one in which participants never meaningfully encountered it.

It is also noteworthy that the greatest loss of participants occurred before the stage requiring more active involvement. This may indicate that future educational interventions focused on testicular cancer should be designed in ways that are even more discreet, simple, and integrated into participants' everyday environment. Potentially more acceptable approaches may include brief digital interventions, micro-educational content, educational messages embedded within routine university settings, or formats that minimize the need for explicit registration or public disclosure of interest in intimate health topics (10, 12, 23, 24).

The observed association between TSE practice and higher optimism also warrants cautious interpretation. Although the finding does not support causal inference, it may suggest that engagement in preventive behavior is linked to psychological resources or a broader style of functioning. If such an association is confirmed in future studies, it may indicate the need to account for psychological factors when designing educational interventions targeted at young men (18, 21). Due to the small sample size, these findings should be interpreted with caution and considered exploratory.

Overall, the study shows that failure to achieve high participation and retention does not diminish the value of the project (2, 13, 19, 24, 25). On the contrary, it provides important information about the practical limits of intervention feasibility in academic settings and about barriers that should be taken into account in future public health efforts. Feasibility assessment makes it possible to identify which elements of a program require modification before more complex or broader preventive interventions are implemente implemented. This observation is consistent with behavioral models that emphasize the intention–behavior gap in health-related actions.

The findings suggest that preventive interventions addressing testicular cancer in young men should be designed to ensure a high level of anonymity, simplicity of messaging, and low-threshold access (2, 12, 13, 23, 25). Interventions that require direct sign-up, open expression of interest in intimate health topics, or greater time commitment may encounter limited acceptability. From a practical standpoint, it seems justified to test more flexible and discreet forms of education, including short online interventions, mobile educational resources, and solutions embedded in participants' everyday environments. From a practical perspective, future interventions targeting young men should prioritize low-threshold access, anonymity, and integration into routine environments, rather than relying on voluntary, multi-stage participation formats.

Future research should include the possibility of monitoring actual exposure to educational materials and should attempt to assess the reasons for disengagement from subsequent stages more directly. It would also be valuable to compare different intervention formats in terms of their acceptability and their influence on participant engagement. Particularly important may be the evaluation of approaches that minimize embarrassment and allow participation without requiring direct disclosure of interest in intimate health issues (2, 12, 13, 24).

### 4.1 Strengths and limitations

A major strength of the study was that the project was conducted under real-world conditions in an academic setting, which made it possible to assess not only interest in testicular cancer prevention but also the practical feasibility of a multi-stage intervention. Another strength was the combination of face-to-face and online strategies, as well as the use of a unique participation code that enabled pseudonymization and the planned linkage of responses across study stages.

The main limitation was the inability to monitor participants' use of the online educational materials, which made it impossible to assess the actual reach of this intervention component. Another important limitation was the very small number of respondents who participated in the final stage of the study, along with the complete lack of participation in the practical workshops, which restricted the possibility of evaluating changes over time and of interpreting the intervention trajectory more fully.

It should also be acknowledged that the detailed analyses were conducted on a subset of participants with complete data and that the study was carried out in a single academic setting, which may limit the generalizability of the findings. At the same time, these limitations are themselves informative from a feasibility perspective, as they reflect the real-world barriers to engagement in preventive interventions.

An additional limitation is the lack of information on individuals who declined participation, which prevents comparison between participants and non-participants and limits the assessment of potential selection bias.

## 5 Conclusions

The implementation of a multi-stage testicular cancer prevention program in an academic setting revealed substantial challenges in maintaining participant engagement across subsequent stages of the project. The availability of educational activities alone did not appear sufficient to ensure participation in workshops or completion of the follow-up stage within this study setting.

The findings indicate a need for interventions that are better tailored to the preferences and barriers characteristic of young men, with greater emphasis on anonymity, lower-threshold access, and formats that are less psychologically demanding.

These findings highlight implementation-related challenges that should be considered when designing future preventive interventions targeting young men.

## Data Availability

The datasets presented in this article are not readily available because None. Requests to access the datasets should be directed to agozdzialska@uafm.edu.pl.
